# Enabling single-site laparoscopy: the SPORT platform

**DOI:** 10.1007/s00464-018-06658-x

**Published:** 2019-01-08

**Authors:** Barbara Seeliger, Michele Diana, Jelle P. Ruurda, Konstantinos M. Konstantinidis, Jacques Marescaux, Lee L. Swanström

**Affiliations:** 1 IHU-Strasbourg Institute of Image-Guided Surgery, 1, place de l’Hôpital, 67091 Strasbourg Cedex, France; 2 Department of Surgical Oncology, University Medical Center Utrecht, Utrecht, Netherlands; 3 Department of General, Bariatric, Laparoscopic and Robotic Surgery, Athens Medical Center, Athens, Greece; 4 Division of GI/MIS, The Oregon Clinic, Portland, OR USA

**Keywords:** Single access, Single-port surgery, Robotic surgery, Single-site, SPORT™ Surgical System, Laparoscopy

## Abstract

**Background:**

The Single Port Orifice Robotic Technology (SPORT) Surgical System by Titan Medical Inc. is designed to overcome the inherent challenges of minimally invasive single-access procedures. The aim of this preclinical study was to evaluate the feasibility of various digestive surgery procedures using this novel surgical robotic platform.

**Methods:**

A total of 12 minimally invasive procedures were performed on six pigs (5 cholecystectomies, 3 Nissen fundoplications, 1 splenectomy and 1 hepatic pedicle dissection) and on one human cadaver (1 cholecystectomy and 1 Nissen fundoplication), by four laparoscopic surgeons. The usability of the device was assessed by means of the modified objective structured assessment of technical skills (OSATS) score that was calculated and analyzed by two independent observers on the recorded videos. Surgeon feedback and recommendations were systematically recorded.

**Results:**

All procedures were successfully completed with the SPORT system. In general, surgeons reported to appreciate the intuitive interface and controls, the high-resolution 3D imaging, the dexterity of the end-effectors, and the ergonomic open control platform. Some features requiring optimization were also identified. The modified OSATS score demonstrated a learning curve effect for all device-related tasks.

**Conclusions:**

A variety of abdominal procedures could be safely completed with the current SPORT prototype, in the preclinical setting. This preliminary feasibility experience is promising and encourages further development of single-port robotically assisted surgery.

**Electronic supplementary material:**

The online version of this article (10.1007/s00464-018-06658-x) contains supplementary material, which is available to authorized users.

Laparoscopy has become the gold standard for most abdominal surgical procedures. With the goal of further reducing surgical trauma, there have been many attempts to reduce the size or number of ports. In particular, single-port or single-site laparoscopy (laparoendoscopic single-site surgery (LESS), single incision laparoscopic surgery (SILS), etc.), while never achieving any widespread adoption, has a persistent and devoted group of followers and there is clinical evidence supporting at least a cosmetic benefit to the approach [1]. However, approaches using a single port are technically much more demanding due to crowding of instruments, minimal triangulation, and clashes between instruments and the camera. Due to these poor ergonomics and the resulting steep learning curve, single-port laparoscopic surgery has never been widely accepted by the surgical community in spite of cosmetic benefits to patients. Robotic surgery has the potential to correct the disadvantages of SILS and offer the ergonomic and performance advantages of multiport robotic systems [2, 3]. Intensive and continuous training is necessary for proficiency in minimally invasive surgery. The more complex a surgical procedure is, the bigger is the expected benefit of robotic assistance [4]. The combined use of minimal access surgery with robotic technology contributes to overcome the inherent limitations of single-port and reduced-port laparoscopy such as inadequate triangulation, poor retraction, lack of wrist articulation, clashing of instruments, poor bedside assistant access, and ergonomic discomfort [3, 5]. Robot-assisted surgery is increasingly performed, and robotic single-site and reduced port surgery remains a current topic [6–11]. In the rapidly evolving field of robotic surgery, various systems are currently being developed to reduce the cost and operating room (OR) footprint of current commercial systems and to enhance ergonomics, tactile feedback, and operating room interaction [3]. Providing a user-friendly open surgeon console and dexterity in performing microsutures, the SPORT™ Surgical System manufactured by Titan Medical Inc. was reported to be a promising device to promote LESS surgery [4]. The aim of this study was to assess the safety and feasibility of single-port and reduced port robotic abdominal surgery with the current prototype single-port SPORT™ Surgical System in a preclinical setting.

## Materials and methods

### Surgeons

The operating expert digestive surgeons (3 consultants and 1 PGY10) regularly carry out minimally invasive digestive procedures. They had variable levels of experience performing robotically assisted surgery (ranging from preclinical training to regular clinical practice). All were naive to the SPORT robotic system. All surgeons were allowed to familiarize with the system in a 30 min dry lab session based on Fundamentals of Laparoscopic Surgery (FLS) manual skills training modules.

### Animals

Six Large White pigs (*Sus scrofa domesticus*) were used in this experimental study, as part of the protocol EVOLVE SPORT (EValuation Of the Learning curVE Of Single Port Orifice Robotic Technology) in the framework of a superordinate experimental protocol (ETICA, ExperimenTations for Innovative deviCes or Approaches), which received full approval from the local Ethical Committee on Animal Experimentation under the reference number 38.2015.01.069, and from the Ministry of Superior Education and Research (MESR) under the reference number 2,015,092,210,412,678 v4 APAFIS#1830. All animals used in the experimental laboratory were managed in compliance with French laws for animal use and care, according to the directives of the European Community Council (2010/63/EU) and the ARRIVE guidelines [12]. Pigs were fasted for 24 h before surgery with free access to water. Premedication using intramuscular injection of 50/50 mg tiletamine/zolazepam and 120 mg azaperone was administered 1 h before surgery. Induction was achieved using intravenous administration of 100 mg propofol combined with 50 mg rocuronium bromide. Anesthesia was maintained with 2% isoflurane. At the end of the experimental protocol, pigs were sacrificed by an over-therapeutic dose of isoflurane (5%) during 10 min, followed by an intravenous injection of Pentobarbital (Exagon® 40 mg/kg). Animals were observed between 10 and 15 min to verify their death before their transfer into the freezer.

### Cadaver

The fresh frozen male human cadaver torso was provided by MedCure Inc., Cumberland, USA, then thawed on site.

### Technology

**Fig. 1:**
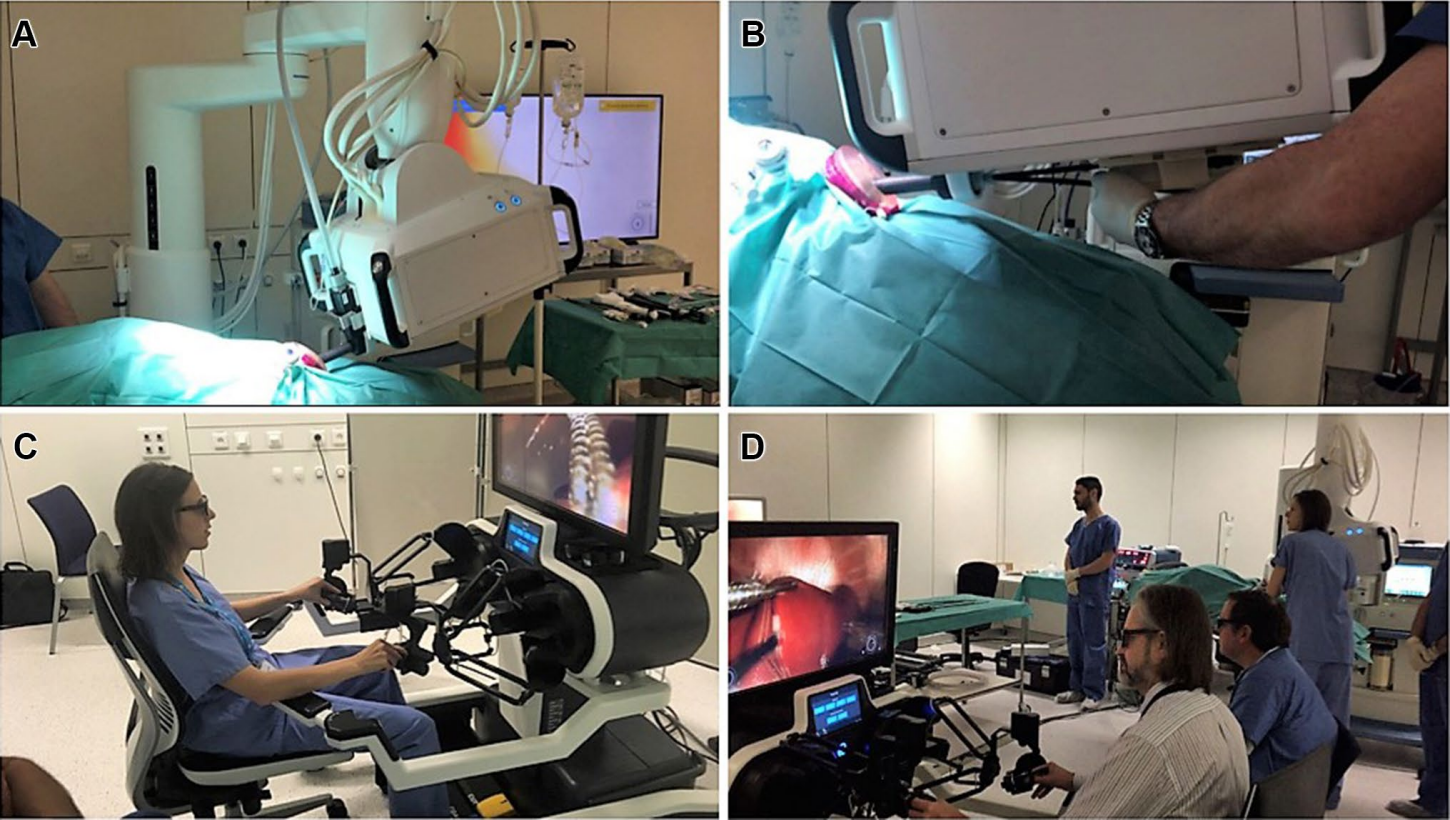
Operating room set-up for a Nissen fundoplication in the porcine model. **A** Patient cart with a single support boom for the CU, which is docked to the CIT. **B** Insertion of the two surgical instruments via the CIT. **C** Surgeon position at the workstation. **D** The open surgeon workstation design allows direct interaction in the operating theatre

The SPORT Surgical System is a single-incision robotic platform, which features multi-articulated instruments with single-use replaceable tips, high-definition 3D visualization with a flat-screen monitor, an ergonomic open workstation, and a single-arm mobile patient cart. The surgeon workstation and the patient cart are the two main components of the system. The patient cart has a single support boom suspending the central unit (CU). It is draped in sterile conditions to be used in the operating field, can be flexibly positioned near the OR table and adjusted using the brake handles (Fig. 1).

**Fig. 2:**
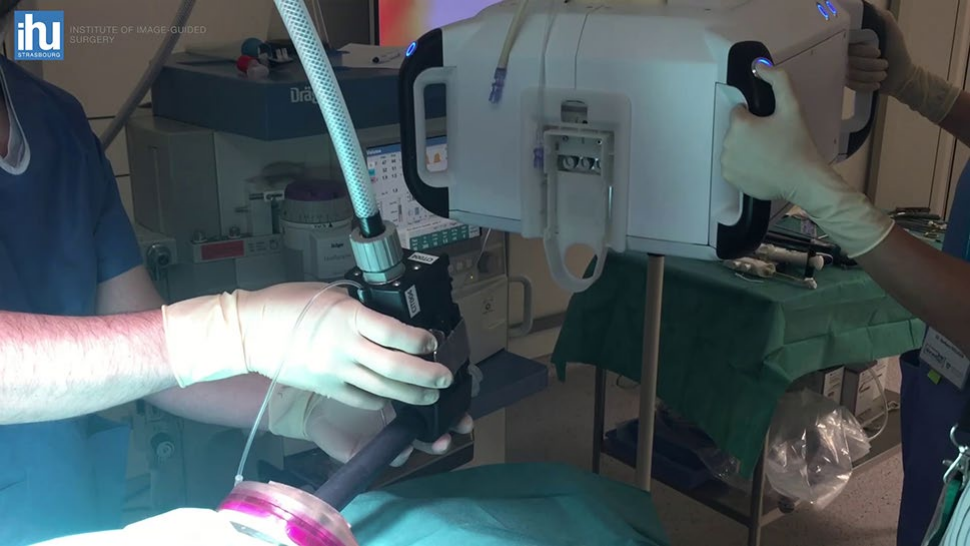
Docking procedure: the CIT is introduced through the gel access port and connected to the CU

In the docking process, a 25-mm-diameter camera insertion tube (CIT) made up of a high-definition 3D camera and light source is connected to the CU **(**Video 1 in the multimedia, Fig. 2 in the text version), and two 8 mm diameter multi-articulated instruments can be inserted through the CIT (Fig. 1).

Ancillary equipment (i.e., lumen seals, instrument boots, integrated lens cleaner) helps to maintain capnoperitoneum during instrument exchanges, to cover the articulating instrument architecture to provide electrical insulation, and to clean the camera lens when soiled.

The surgeon workstation is a complete open console equipped with a 32″ 3D high-definition flat-screen monitor, allowing direct interaction with the operating room staff. The surgeon is seated in an ergonomic position at the console, which can be individually adjusted, and operates the instruments using a natural handle interface of finger-tip with clutching akin to open surgery (Fig. 1).

During the surgeons’ introductory dry lab experiences, the optimal working distance between the SPORT system’s CIT and the target zone was measured to be 19 cm, to provide optimal instrument motion range in the camera-provided field of view. A closer distance to the target zone entailed reduced working space and functionality and when reaching the boundaries of instrument motion range, instrument grip force was reduced. As a result, a learning curve was required to gain familiarity with the motion range while keeping the necessary grip strength for tissue and needle manipulation.

### Surgical procedures

Ten digestive surgery procedures were performed in the living animal model (five cholecystectomies, three fundoplications, one splenectomy, and one hepatic pedicle dissection). Two additional procedures (cholecystectomy and fundoplication) were completed in the human cadaver specimen. Animals as well as the human torso were placed in supine reverse Trendelenburg position, prepped and draped prior to incision. The primary abdominal access site was chosen according to the optimal working distance as determined in the dry lab experience. Abdominal access was achieved with a single 30 mm incision with a self-retaining retractor (Alexis Wound Retractor, Applied Medical) attached to a gel access port (GelPOINT, Applied Medical). After establishing the capnoperitoneum, the CIT with the camera in a non-deployed position was introduced into the abdomen via the gel port. The CIT was then connected to the CU, the camera was deployed, and the instruments were inserted. During the procedures, on-site engineers adjusted the grip strength of the multi-articulated instruments upon surgeons’ needs. For some procedures, an additional assistant port was placed for insertion of surgical instruments such as retractors.

### Evaluation

All procedures were recorded with combined internal and external views for performance analysis as well as evaluation of ergonomics, communication in the theatre and structured assessment of the technical skills. The modified Objective Structured Assessment of Technical Skills (OSATS) Score (Table 1) as published by Willems *et al*. [13] was chosen as a scoring method, since it is a validated assessment tool for objectively grading overall technical proficiency in surgery. The OSATS scores were calculated and analyzed by two independent observers using both internal and external views to assess surgeons’ individual performances. Modified OSATS skills scores were analyzed using Spearman’s rank-order correlation to assess potential learning curves individually. At the end of each day, a systematic debriefing procedure was carried out.

**Table 1:** Modified Objective Structured Assessment of Technical Skills Score as published by Willems *et al*. [13] used for postprocedural analysis. For cholecystectomy, skills 6 and 8 were adapted (use of clip and clip placement, respectively) and skill 7 was eliminated due to the absence of suturing

Skill	Score		
	1 2	3	4 5
1. Respect for tissue	Frequently used unnecessary force on tissue or caused damage by inappropriate use of instruments	Careful handling of tissue but occasionally caused inadvertent damage	Consistently handled tissues appropriately with minimal tissue damage, no rough handling
2. Time and motion	Many unnecessary moves	Efficient time/motion but some unnecessary moves	Clear economy of movement and maximum efficiency
3. Instrument handling	Repeatedly makes tentative or awkward moves with instruments	Competent use of instruments although occasionally appeared stiff or awkward	Fluid moves with instrument and no awkwardness
4. Flow of operation	Frequently stopped operating and seemed unsure of next move	Demonstrated some forward planning with reasonable progression of procedure	Obviously planned course of operation with effortless flow from one move to the next
5. Knowledge of specific procedure	Deficient knowledge; needed specific instructions at most steps	Knew all important steps of operation	Demonstrated familiarity with all aspects of operation
6. Use of suture material or clip	Crossed and tangled suture material, misplaced clips	Minimal tangling	Excellent suture material or clip control
7. Knot quality	Mainly sloppy knots	Half of the knots are square	Mainly square knots
8. Suture/clip placement	Imprecise inconsistent suture/clip placement	Good suture/clip placement, and consistency with some variability	Precise consistent suture/clip placement
9. Back-walling of a suture / clip	Yes □ -10 points	No □	
10. Comfort rating of the surgeon	Very uncomfortable/much tremor	Some discomfort/increase of tremor	No discomfort/minimal tremor

## Results

All procedures were successfully completed. Intraoperative blood loss was negligible in the live animal model. Three cholecystectomies were performed via the single access by one surgeon. Two more surgeons performed a cholecystectomy each, one with an additional retraction instrument, and one in a fundus-down technique due to difficulty exposing the cystic duct. In one pig, the hepatic pedicle was chosen for anatomical dissection. Using an internal liver retractor (Surgical Perspective, Strasbourg, France), one Nissen fundoplication was accomplished through a pure single-port technique, while the other two fundoplications were performed using an additional trocar to insert a liver retractor. A splenectomy was accomplished by two surgeons, one dissecting and dividing the superior vascular supply, one dissecting and dividing the inferior vascular supply and extracting the spleen through the single incision using a specimen bag. Clip application and the insertion of a specimen extraction bag were performed through an additional trocar inserted in parallel to the CIT, through the single abdominal access.

On the 62-year-old male torso with a body mass index of 14, transumbilical access served to perform a cholecystectomy and a Nissen fundoplication with transhiatal mobilization of the esophagus, using an additional port for liver retraction.

**Fig. 3:**
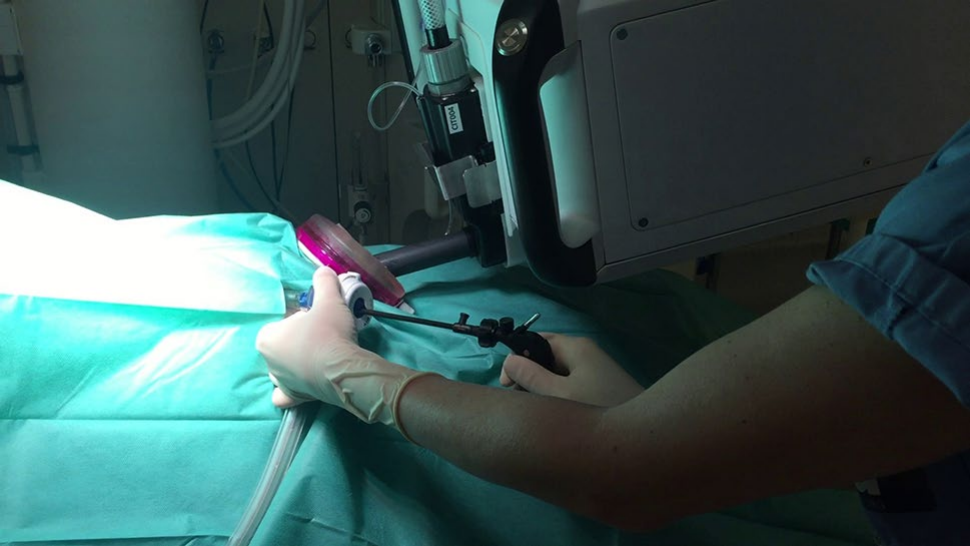
Assistant introducing a standard laparoscopic instrument through an additional assistant port. The port distance to the CIT and CU allows for a large motion range

The small OR footprint allowed for a flexible positioning of the patient cart and an easy docking procedure, as well as for the table-side assistant to easily adapt the CU angle to any new target region. The complete docking procedure including insertion of the CIT through the gel access port, CIT connection to the CU, and insertion of the desired surgical instruments took approximately one minute (Video 1 in the multimedia, Fig. 2 in the text version). With optimal port placement, the SPORT system provided sufficient workspace for surgical procedures performed in different abdominal quadrants. Additionally, in the absence of camera lens fogging, removal of the CIT was not required during the whole surgical procedures. If necessary, the lens wash feature provided lens cleaning intra-abdominally, being activated by the assistant on the CU or on the surgeon’s secondary screen. As the surgeon does not control CIT movement from the workstation, the table-side assistant was responsible for repositioning maneuvers. The table-side assistant was also responsible for retraction or insertion of accessory devices such as suction-irrigation devices or staplers, either through the gel port single-site access or via a secondary port. Using a secondary port, the assistant had a large workspace as compared to multi-arm robotic set-ups (Fig. 3). Due to the patient cart design, there were no external moving parts during the functional use of the surgical system, which markedly improved the assistant’s comfort and workspace. When placing the assistant port through the single-site access, the assistant’s motion range was limited by collisions with the CU handles and box when working in parallel to the CIT. The assistant’s motion range can further be enhanced by either increasing the distance between the incision and the CU (lengthening the CIT) or by miniaturizing the CU size. These considerations will be implemented into the design of the subsequent prototype version.

Systematically recorded surgeon evaluation of the platform using a visual analog scale ranging from 0 to 10 was carried out. The mean scores for different system components revealed appreciation of the ergonomic open control platform (8,5 +/- 1), the intuitive interface and controls (8,25 +/- 0,5), the dexterity of the end-effectors (7,63 +/- 0,48), and the high-resolution 3D imaging (6,75 +/- 1,5).

The modified OSATS Score (Table 1) allowed to objectively evaluate each surgeon’s performance. The two observers’ scores showed congruence, thereby validating the choice of this assessment tool. Using Spearman’s rank-order correlation, a learning curve-related increase in the technical skills score was observed for surgeons 1, 3 and 4 (r > 0.77 for skills 1, 2, 3, 4 and 10). These skills (1, respect for tissue; 2, time and motion; 3, instrument handling; 4, flow of operation, and 10, comfort rating of the surgeon) are correlated to familiarity with the robotic system and experience. The other skills (knowledge of specific procedure, use of suture material, knot quality, suture placement and the absence of suture back-walling) did not show any variation over the successive procedures, indicating a general understanding of surgical procedure planning and suture/clip placement for each surgeon.

**Fig. 4:**
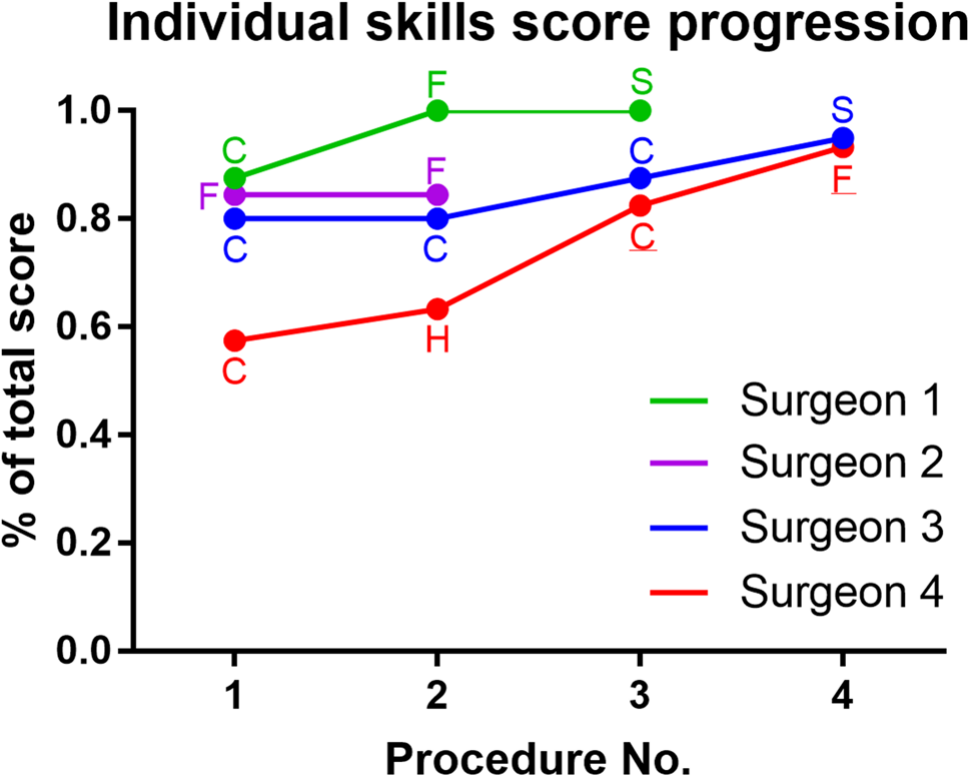
Individual analysis of modified OSATS score revealed an overall learning curve effect represented by an increasing percentage of total score with every performed procedure. Procedures are represented as follows: C (cholecystectomy), F (fundoplication), H (hepatic pedicle dissection), S (half splenectomy). Cadaveric procedures are underlined

For surgeons 1, 3 and 4, the subjectively reported confidence gain with each performed procedure corresponded to reaching a maximum comfort rating OSATS score at the last one performed (3rd, 4th and 4th procedure, respectively). Surgeon 2 obtained a relatively high score (38/45 points) in both performed procedures. However, the sample number was too small for correlation analysis. The individual procedural performance is shown as percentage of total modified OSATS score for each surgeon’s procedures in Fig. 4. All surgeons perform robotic procedures to a varying degree. We surmised that a higher degree of robotic experience could well predict a better performance using the SPORT Surgical System. However, the highest and lowest starting scores were obtained by the most experienced robotic surgeons. This finding underlines the learning curve effect and the importance of training necessary to achieve proficiency in any new surgical system.

## Discussion

This paper reports on the first procedures performed with a new robotic system for single site surgery. All procedures performed were completed without intraoperative complications or conversions.

The unique design of the patient cart with a single-arm mobile CU allowed for a quick and easy docking procedure. The intuitive control of the two multi-articulated instruments provided sufficient surgical dexterity. In some cases, to facilitate retraction without the need for an additional assistant port, a third arm or internal retractors were estimated helpful. The camera controls solely accessible on the hand controls worked adequately, but could have been better positioned from an ergonomic standpoint. In the current prototype version that we used, camera resolution and white balance properties are still under development and will be improved in the next version. So far, brighter images will be required, since activating the zoom function to focus on distant working areas led to a significant loss of brightness. The camera system would benefit from a larger range of motion to minimize CU adjustments and to allow a closer approach to target organs and achieve a critical view of safety. In addition, this would allow surgeons to increase the workspace in the abdominal cavity. Enlarging the distance between the CU and the abdominal access would improve the currently limited working range of the assistant in the single-access setting.

Current regulatory pathways require specific capabilities of robotic surgical instruments. As evaluated in postprocedural video analysis, each surgeon performed the critical tasks including tissue grasping and retraction, dissection, cutting, monopolar electrosurgery and suturing with the necessary dexterity and precision, whether in single-incision procedures or using an additional assistant port.

As with any new surgical technology, thorough testing can be complex and time-consuming. Robotic surgery has come a long way and the possibility to perform complex procedures through a single incision appears more valid than ever before. Basic requirements include excellent visualization in high-definition 3D, easy access to the body with minimal trauma, and robust articulating arms to approach the target tissue from multiple angles. We trialed a new robotic single-port platform, which has proven to solve complex issues related to instrument strength, natural instrument movement and access to any desired patient anatomy. With the availability of an advanced prototype, we performed complex abdominal surgical procedures in vivo in a porcine model and in a human cadaveric model to assess feasibility and to explore the limits of the system.

While the investigators only had minimal training and practice, the current prototype version allowed an intuitive control of the robotic laparoscopic instruments within the operative environment in this study. Nevertheless, like with all advanced instrumentation, a learning curve is apparent and surgeons interested in this technology would need structured training and practice. A set of core surgical skills simulation modules for the SPORT surgical platform has just been completed. Two of the surgeons had the opportunity to trial the simulator and felt like it would further reduce the learning curve.

The previous limitations of laparoscopic single-port surgery, with conflicting instruments and an intimidating learning curve, or even current robotic single-port surgery involving the crossing of two non-articulating robotic arms within the single-site port, are overcome with this system design. The surgeons benefited from improved ergonomics, within an open control station, high-definition 3D imaging, and enhanced dexterity. Table 2 lists the potential benefits of this surgical system as compared to standard laparoscopic single-port surgery.

**Table 2:** Potential benefits of the SPORT surgical system as compared to conventional laparoscopic single-port surgery (Disadvantage: -, Advantage: +)

	Conventional laparoscopic single-port surgery	Sport surgical system
*Ergonomy*		
Surgeon	−	+
Assistant	−	+
*Image quality*		
5 mm laparoscope vs. 3D imaging system	−	+
Camera insertion towards target zone	+	−
Camera exchange (0°, 30°)	+	−
*Instruments*		
Multi-articulated instruments	−	+
Specialized (vessel sealing, stapling)	+	−
*Conflicts*		
Instrument clashing	−	+
Instrument / camera conflict	−	+
OR Footprint	+	−

Although the SPORT Surgical System is a platform which is still in the final stages of development, it is a promising new opportunity to broaden the applications of ergonomic single-site surgery. Its application in other types of minimally invasive surgery is not yet known. Procedures related to SILS such as transoral or transanal access surgeries present possible future applications for this platform.

A recent meta-analysis showed better outcomes in terms of cosmesis, body image and postoperative pain for single-port laparoscopic cholecystectomy [14]. The risk of incisional hernia was found to be significantly higher (4.0 vs. 1.1 percent, p = 0.03) in single-port compared to conventional laparoscopic cholecystectomy. However, the authors discuss fascia closure technique and refer to a recent retrospective study, where no incisional hernia occurred in 500 consecutive single-port laparoscopic cholecystectomies when fascia closure was performed with two figure-of-eight knots instead of one [15]. However, the true benefits of single-port laparoscopy vs. conventional laparoscopy or robot-assisted laparoscopy remain unsolved.

Another persisting issue with robot-assisted surgery is the added cost as compared to laparoscopy. As we trialed a non-commercially available system, the actual procedure cost is still unknown. The manufacturing company expects that when the system becomes commercially available, it will offer cost savings as compared to currently available robotic platforms.

Having safely completed a variety of digestive surgery procedures with the advanced SPORT prototype in the preclinical setting, this preliminary feasibility experience is promising and encourages further development of single-port robotically assisted surgery.

## Electronic supplementary material

Below is the link to the electronic supplementary material.

Video 1 Introduction of the CIT through the gel access port, connection of the CIT to the CU, and insertion of two multi-articulated instruments. (MP4 55915 KB)
